# Ambulatory cataract surgery centre without perioperative anaesthesia care: a prospective cohort study

**DOI:** 10.1038/s41598-021-87926-0

**Published:** 2021-04-15

**Authors:** Quentin Duroi, Jean-Marie Baudet, Maxime Bigoteau, Malek Slim, Tiphanie Pichard, Pierre-Jean Pisella, Raoul Kanav Khanna

**Affiliations:** 1 Department of Ophthalmology, Centre Hospitalier Universitaire Régional de Bretonneau, Bretonneau University Hospital of Tours, 2 Boulevard Tonnellé, 37000 Tours, France; 2 Department of Ophthalmology, Centre Hospitalier Jacques Coeur, Bourges, France; 3 INSERM 1253 iBrain «Neurogénomique & Physiopathologie neuronale», Tours, France

**Keywords:** Lens diseases, Surgery

## Abstract

This study aims to evaluate the safety and patient satisfaction of a fast-track procedure for cataract surgery under topical anaesthesia without perioperative anaesthesia care. This is a prospective single-centre study including all cataract procedures in the Centre Ambulatoire de la Chirurgie de la Cataracte at the Hospital of Bourges between May and August 2018. Procedures were performed under topical anaesthesia without the presence of a nurse anaesthesiologist or anaesthesiologist, the patient had not fasted, and no peripheral venous line was placed. Only heart rate and oxygen saturation were monitored intraoperatively with pulse oximetry. Incidence and nature of intraoperative adverse events and surgical complications were recorded. Patient satisfaction was assessed using the Iowa Satisfaction with Anaesthesia Scale (ISAS). In total, 651 cataract surgeries were performed among which 614 (94.3%) were uneventful. Thirty (4.6%) intraoperative adverse events and 8 (1.2%) surgical complications were recorded. All surgeries were successfully completed. No medical emergency team intervention or hospital admittance was encountered. The mean ISAS score was 5.7/6, indicating high patient satisfaction. Cataract surgery in an ambulatory cataract surgery centre without perioperative anaesthesia care is a safe procedure with high patient satisfaction for screened patients. Anaesthesia ressources are scarce and may be more beneficial to more complex ophthalmic or non-ophthalmic surgeries.

## Introduction

Cataract surgery is the most commonly performed surgical procedure worldwide. The generalization of phacoemulsification and the progress of less and less invasive surgical techniques, associated with shorter operating times, have gradually led to a reduction in perioperative anaesthetic care. Currently, topical anaesthesia with eye drops is the most widely used method with comparable efficacy to other anaesthesia methods^1–4^. Due to its ease of implementation, topical anaesthesia allows a rapid turnover of patients in the operating room, while avoiding the complications of locoregional anaesthesia^2–4^. Moreover, in recent studies, the need for an anaesthetic procedure during cataract surgery under topical anaesthesia has been shown to be rare^5–8^.

The evaluation report of the French National Authority for Health (HAS) of July 2010 on the "Conditions for performing cataract surgery" paved the way for a simplification of procedures^9^. This report states that a pre-anaesthetic consultation is mandatory in France in the case of general or locoregional anaesthesia, but not mandatory in the case of topical anaesthesia. It also specifies that the use of topical anaesthesia does not require the presence of an anaesthesiologist in the operating room. It is recommended for patient comfort and safety, however, that a referring anaesthesiologist be available when needed.

In France, some teams have begun to offer simplified care for patients undergoing cataract surgery under topical anaesthesia without a systematic preoperative anaesthetic consultation or without an anaesthesiologist present in the operating room^10–13^. This change in practice is in response both to a lack of personnel available to provide conventional anaesthetic management in ophthalmology (preoperative consultation, intraoperative monitoring) and to an increased demand for care given the aging of the population and increased life expectancy.

**Figure 1:**
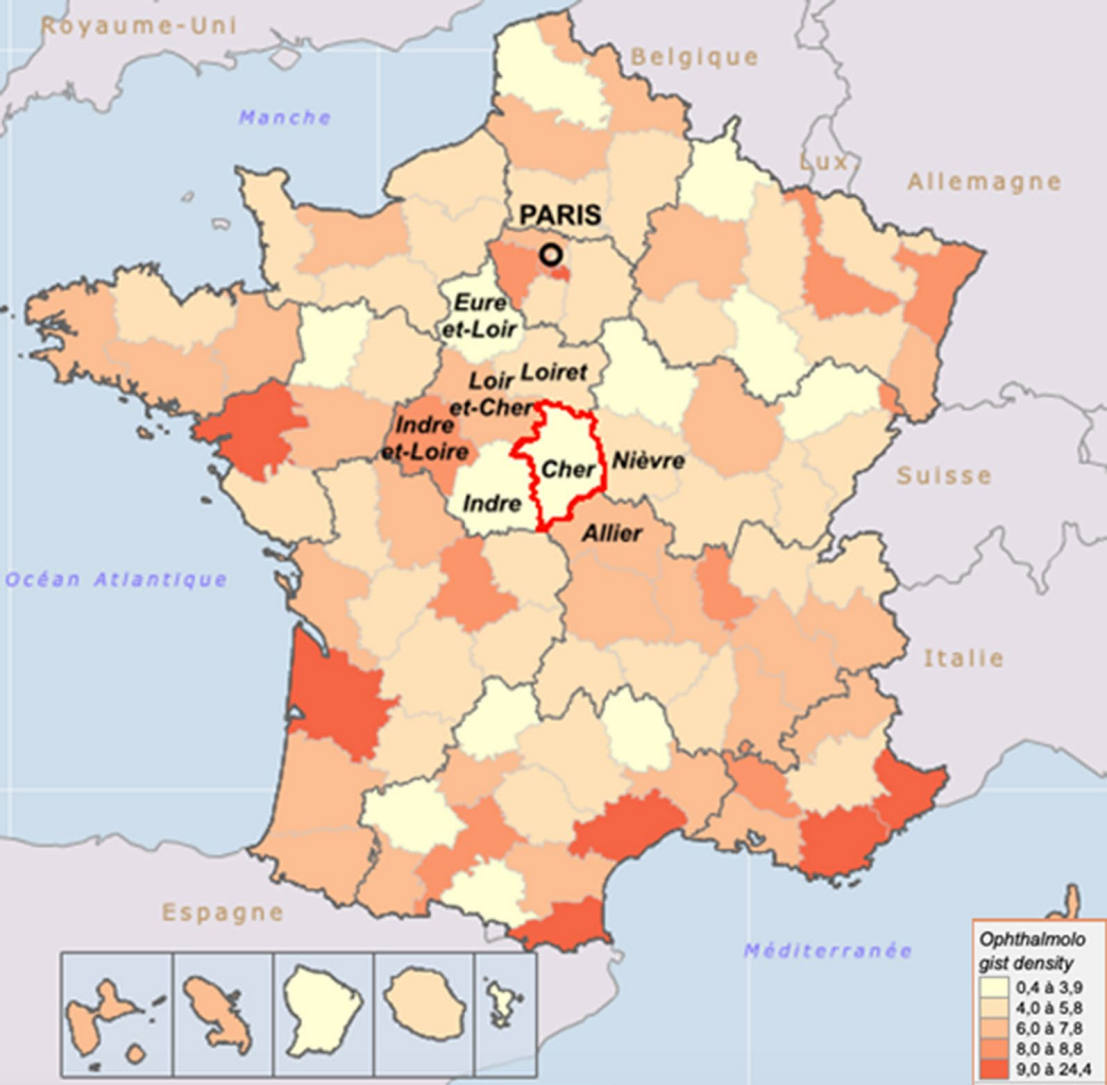
Cartography of ophthalmologist density (according to area) expressed per 100,000 inhabitants (extracted from https://demographie.medecin.fr/mobile.php by MB).

The Cher area, which capital city is Bourges, is a medical desert (3.2 ophthalmologists per 100,000 inhabitants in 2018, that is to say 10 ophthalmologists; Fig. 1). Since 2015, at the Centre Ambulatoire de la Chirurgie de la Cataracte (CACC) of the Centre Hospitalier Jacques Coeur de Bourges (CHB), eligible patients are operated for cataract surgery on an outpatient basis, under topical anaesthesia, in a dedicated autonomous operating room, without pre-anaesthetic consultation and under the exclusive supervision of the surgical team, without perioperative anaesthetic care. In a previous study, we highlighted the beneficial epidemiological effect of such a structure to improve care in a medical desert^14^.

The main objective of this prospective study was to evaluate the incidence of intraoperative complications and adverse events during cataract surgery in the CACC. The secondary objective was to evaluate patient satisfaction.

## Materials and methods

### Study design and population

This prospective single-centre observational study included all patients operated on for cataracts in the CACC at the CHB between May and August 2018. This study was performed using anonymized data and approved by the Ethics Committee of the University Hospital of Tours, France (registration number: 2018034) and the French national commission for information technology and civil liberties (registration number: 2018_062). All subjects provided informed written consent. This study was conducted in accordance with the Jardé law as well as the ethical principles stated in the Helsinki declaration.

### Surgical facility

From September 2015, the new ophthalmology department of the CHB, a public general hospital, opened its doors in an independent building. Including on one side the consultations unit and on the other the CACC with an autonomous procedure room dedicated to cataract surgery, allowing most cataract surgeries to be outsourced in the CACC (93.6% in 2018 according to Bigoteau et al. 2020^14^). In agreement with the anaesthesia and surgical teams, patient safety was the responsibility of the CACC team, consisting of a surgeon, a resident and two operating room nurses. In the event of emergency, the Emergency Medical Assistance Service (SAMU) of the CHB could be contacted at any time to respond. The CACC is equipped with an emergency cart, a defibrillator and the necessary drugs for reanimation. CACC staff are regularly trained in first aid techniques and cardiopulmonary reanimation.

### Patient track from preoperative to postoperative assessment

The patient track within the CACC is presented in Supplemental file 1 and the main elements of the care pathway are summarized in Table 1.

**Table 1:** Patient care from the preoperative consultation until the day after the intervention.

	Location	Procedure
Preoperative consultation	Consultation unit	Ophthalmic examination
		Medical and surgical history
		Allergies
		Ongoing treatments
		Eligibility for cataract surgery in the CACC
		Information about the chosen care pathway
On the eve of the operation		Call from the healthcare team
		Preoperative instructions, hygiene rules, schedules and transport arrangements
On the day of surgery	At home	The patient is not fasted
		Taking usual medication
		Anxiolytic premedication with alprazolam 0.25 mg
	In the CACC	Admittance
		Check of the compliance with preoperative instructions
	Locker room	Storage of personal belongings
	Waiting room	Dilatation (MYDRIATICUM, NÉOSYNÉPHRINE)
		Blood pressure measurement
	Transfer airlock	Seated on a stretcher chair
		No venous route
	Operating room	Topical anesthesia (OXYBUPROCAÏNE, VISTHÉSIA)
		Pulse oxymetry
		Phacoemulsification procedure
	Post-operative monitoring room	Rest
		Pain assessment
		ISAS satisfaction questionnaire
	Postoperative consultation	Slit-lamp examination
		Postoperative recommendations
	Conviviality room	Snack
		Departure
On the day after the operation		Call of the patient by a nurse

The preoperative screening criteria excluding treatment within the CACC were as follows: bad cooperation, intractable cough, tremor, respiratory or cardiac insufficiency not permitting a supine position, risk of vasovagal response, ocular disease requiring anaesthetic intervention and impossibility of raising patients. If none of these criteria were observed, the patient was eligible for outpatient surgery under topical anaesthesia at the CACC. In this case, no further medical examination was required and the preoperative anaesthesia consultation was not required. Patients were asked to continue their usual medication and to eat or drink normally on the day of surgery. A prescription for anxiolytic premedication with alprazolam 0.25 mg (XANAX) to be taken the day before and the day of the operation was offered to patients.

In case of systolic blood pressure ≥ 180 mmHg and/or diastolic blood pressure ≥ 110 mmHg, the surgeon was warned and could decide to make the patient wait in the waiting room and request a second measurement. If the second measurement remained high, the surgeon could have the nurse administer a single dose of oral antihypertensive drug: nicardipine 20 mg (LOXEN) according to the department's protocol. In the absence of any contraindication after a final measurement, the patient was then admitted to the transfer airlock where they were comfortably seated on the stretcher chair and covered with a warmed sheet. No venous route was placed.

### Surgical procedure

In the operating room, the patient was placed in the supine position, with a free-flow of oxygen under the operating drapes and benefited from simple monitoring by pulse oximetry (measurement of heart rate and oxygen saturation). Topical anaesthesia was administered by instillation of anaesthetic eye drops (OXYBUPROCAINE HYDROCHLORIDE 0.4%, Théa©) then by application of a 2% lidocaine hydrochloride gel (VISTHESIA, Zeiss©) after strict surgical disinfection of the eye with ophthalmic Betadine. The anaesthesia was systematically supplemented at the start of the operation by an intracameral injection of a viscoelastic solution of sodium hyaluronate and 1% lidocaine hydrochloride (VISTHESIA Intracameral). The surgery was performed by phacoemulsification according to the conventional “Divide and conquer” technique (STELLARIS, Bausch & Lomb Incorporated©). Surgery could be performed by the resident accordingly to the senior surgeon’s decision. If necessary, verbal and tactile reassurance were provided by the nursing staff in the operating room.

### Data collection

**Figure 2:**
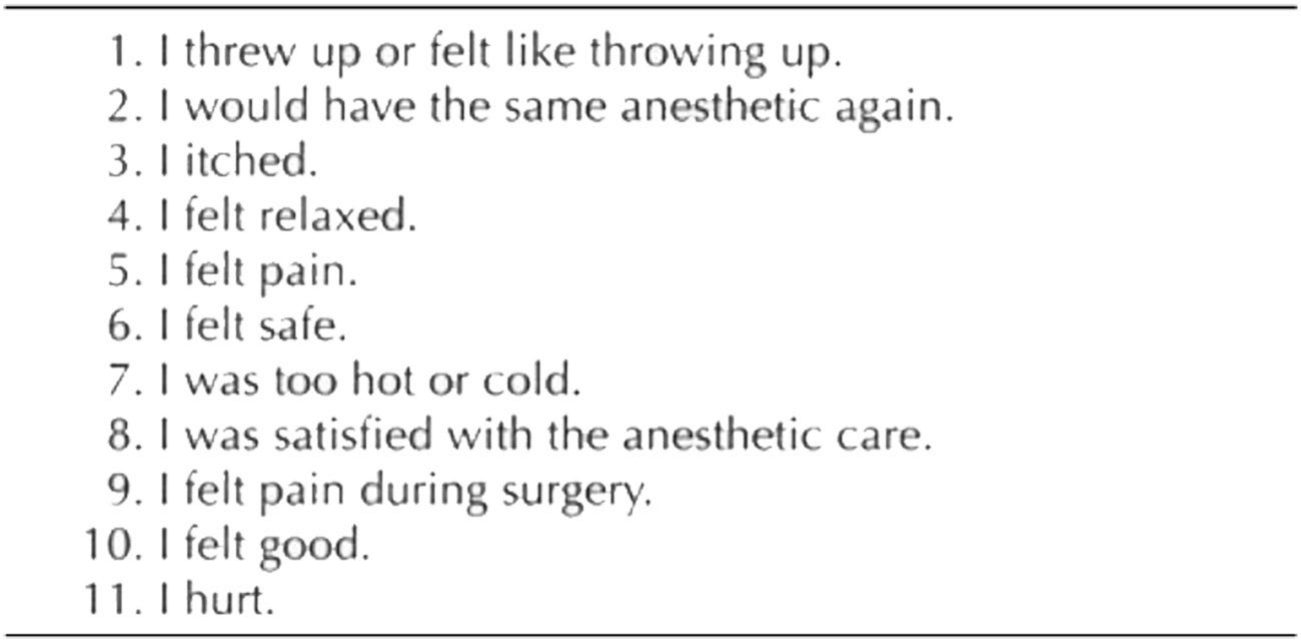
ISAS, Iowa Satisfaction with Anaesthesia Scale^15^ (copyrighted by Franklin Dexter and the University of Iowa Research Foundation). For items 1, 3, 5, 7, 9, and 11, the scores are calculated as follows: + 6, agree very much; + 5, agree moderately; + 4, agree slightly; + 3, disagree slightly; + 2, disagree moderately; + 1, disagree very much. For items 2, 4, 6, 8, and 10, the scores are calculated in the reverse order from the aforementioned items.

Unusual events encountered by the surgeon were noted on the collection sheet by the surgeon or the nurse, either during or at the end of the procedure. We then collected and classified these events into adverse events or surgical complications. The pain felt during the procedure and before leaving the monitoring room was evaluated by a verbal analogue scale from 0 to 10 (replaced by a simple verbal scale in case of significant visual disturbance). If it was the first eye operated on, the patient indicated whether they would agree to undergo the operation again under the same anaesthetic conditions. A simple verbal satisfaction scale between 0 and 10 and the Iowa Satisfaction with Anaesthesia Scale (ISAS)^15,16^ questionnaire were used. ISAS is a tool for assessing patient satisfaction during cataract surgery which has been validated in French (Fig. 2)^17^. It is a written questionnaire including eleven items dealing with pain, sensations and the anaesthetic experience. With the maximum score being 6, satisfaction is considered high for a score above 5.4^18^. This copyrighted questionnaire is the property of Franklin Dexter and the University of Iowa Research Foundation.

### Statistical analyses

Data were collected using Excel software (Microsoft Corporation©). Data collection was double-checked by QD and RKK. Due to the observational nature of our study, no statistical analysis was performed. The quantitative variables were described by the mean ± standard deviation. Qualitative variables were described by number and percentage.

## Results

In total, 651 surgeries (500 patients; 303 women; mean age: 74 years, range: 43–96) were included. Demographic data and comorbidities are described in Table 2. At least one general comorbidity was identified in 36% of cases and 36.2% of subjects had at least two general comorbidities. Anxiolytic premedication by alprazolam 0.25 mg (XANAX) was taken by the patient in 95.7% of cases. One hundred and fifty-one patients (32%) underwent successive surgery on both eyes during the study period. This was a second eye surgery for 312 (47.9%) patients. The overall average duration of patient management in the CACC between admission and return home was 125 ± 32 min (range: 53–280).

**Table 2:** Characteristics of the population.

		Percentage
Patients	500	–
Age (years) (mean ± standard deviation)	74 ± 8	–
Women	303	60.6
**General comorbidity**		
0	139	27.8
1	180	36
≥ 2	181	36.2
High blood pressure	236	47.2
Diabetes mellitus	117	23.4
Cardiovascular disease	130	26
Thyroid disease	20	4
Respiratory disease	14	2.8
Kidney disease	9	1.8

### Preoperative blood pressure assessment

The systolic and diastolic blood pressure values at the first measurement, then at the second for uncontrolled blood pressure, are described in Table 3. No patient was discharged for uncontrolled blood pressure.

**Table 3:** Preoperative blood pressure measurements.

	Normal blood pressure^a^	Initially uncontrolled blood pressure	Initially uncontrolled blood pressure requiring Nicardipine (LOXEN)
N (%)	624 (95.9)	25 (3.8)	2 (0.3)
**First measurement**			
Systolic blood pressure (mmHg)	136 ± 16	182 ± 10	201 ± 12
Diastolic blood pressure (mmHg)	68 ± 12	71 ± 20	86 ± 19
**Second measurement**			
Systolic blood pressure (mmHg)	–	152 ± 15	172 ± 10
Diastolic blood pressure (mmHg)	–	73 ± 26	60 ± 5

### Surgery

The procedures were performed by 7 surgeons: 3 hospital practitioners and 4 specialist assistants. Surgery was performed by a resident in 7.68% of cases (50 procedures). The mean duration of the surgeries was 14 ± 7 min (range: 5–75). No adverse event or surgical complication was found in 614 cases (94.3%). The power of the intraocular lens ranged from − 3 to + 32. Ninety-three custom-made intraocular lenses (14.3%, 9 multifocal, 84 toric) were placed.

### Adverse events and surgical complications (Table 4)

**Table 4:** Adverse events and surgical complications.

	N	Percentage
Adverse events	30	4.6
Agitation/anxiety	16	2.5
Bradycardia (< 60)	8	1.2
Pain	4	0.6
Oxygen desaturation (< 93%)	1	0.15
Nausea	1	0.15
Call to the emergency medical assistance service	0	–
Surgical complications	8	1.2
Iris prolapse	3	0.5
Posterior capsular rupture with anterior vitrectomy	3	0.5
Anterior capsular rupture	1	0.1
Explantation	1	0.1
Posterior lens dislocation	0	–
No implantation	0	–

An adverse event occurred for 30 procedures (4.6%). None of these events led to an interruption of the procedure. No intervention by the SAMU team was necessary. No case of posterior lens dislocation or implantation failure was reported.

### Pain, ISAS score and patient satisfaction

The pain experienced during the procedure was evaluated by patients at 0 out of 10 for 586 procedures (90%). The mean intraoperative pain was 0.2 ± 0.7 (range: 0–6) and the mean pain on leaving the monitoring room was 0.1 ± 0.5 (range: 0–5). Mean patient satisfaction with the intervention was 9.1 ± 0.9 (2–10). The mean ISAS score was 5.66 ± 0.42 (range: 3.3–6). Of the 339 individuals operated on for the first eye, 329 indicated that they would like to have the operation for the second eye under the same anaesthetic conditions (97%).

## Discussion

The procedure described in our study represents a simplified, safe and satisfying care pathway for screened patients eligible for cataract surgery under topical anaesthesia without the following elements: preoperative assessment or blood test, anaesthetic visit, modification of the usual treatments, preoperative fasting, venous line, intravenous sedation.

### Perioperative morbidity and mortality

Although cataract surgery concerns an elderly and often poly-pathological population, the morbi-moratily rate is very low^19^. Though severe complication have been reported (i.e. cardiac arrest^20^ or severe bradycardia with myocardial infarction^21^), these events are extremely rare and the perioperative morbi-mortality is estimated to be between 0.01 and 0.05%^6–8,22^, rates which are similar to the risk in the general population during daily living activities.

### Choice of anaesthesia technique and patient selection

The perioperative anaesthetic care in cataract surgery is still debated and practices vary worldwide^23–26^. Within the CACC, the type of anaesthesia performed is topical anaesthesia. Thus, it is the surgeon's responsibility to decide, for each patient, whether cataract surgery can be performed within the CACC under these conditions. A decision-support screening sheet was designed in partnership with the hospital's anaesthesia team to guide the ophthalmologist. The criteria used are non-binding and the final decision rests with the surgeon, adapting on a case-by-case basis.

The French High Healthy Autority report states that a preoperative anaesthesia consultation is mandatory in the case of general or locoregional anaesthesia whereas it is not always necessary for topical anaesthesia. Performing a systematic preoperative workup before cataract surgery (electrocardiogram and blood test) has shown no benefit in terms of reducing adverse events^27^. Although some patients may be at high risk of complications (e.g. severe diabetes, severe renal dysfunction, severe liver dysfunction), there is good evidence that systematic preoperative blood test prior to cataract surgery does not increase the safety of cataract surgery while increasing costs^27,28^. Regarding the haemorrhagic complications related to antiplatelet and anticoagulant medications, some studies found a significantly higher incidence of subconjunctival haemorrhage but no significant difference in the incidence of complications nor visual outcomes^29,30^. Thus, these treatments are usually not discontinued before surgery. Preoperative fasting does not appear necessary in case of topical anaesthesia and can even be destabilizing for elderly patients^31^. The patients in the current study were instructed to eat normally on the day of surgery and to continue all their usual treatments in order to modify their usual routine as little as possible.

The innovative care pathway set up at the CACC responds to a reasoned use of the available medical resources, while offering patients the appropriate care, adapted and optimized, without unnecessary assessment or therapy. In fact, in a previous study, we showed that this treatment path could be used on a large scale (i.e. 93.6% of cataract surgeries performed in the CACC in 2018) and was not associated with additional costs compared to the conventional operating room (Supplemental file 2).

### Surgery with or without an anaesthesiologist?

Several studies have been published on the safety of a protocol without an anaesthesiologist present in the operating room. The measurement of the rate of adverse events and the need for an anaesthesiologist during surgery are the usual endpoints. Bassett et al. compared, in 211 patients, the intraoperative monitoring entrusted to a specially trained nurse versus an anaesthesiologist in terms of patient safety and satisfaction^32^. No serious complications occurred in the two groups and the satisfaction scores were similar. In a study performed by Rocha and Turner, 538 surgeries were realized under oral sedation without an anaesthesiologist present in the operating room but available on demand^5^. In 5 cases (0.9%) significant changes in blood pressure required the intervention of the anaesthesiologist. They did not report any surgical complications or unplanned admissions to hospital. In the series of 1002 surgeries reported by Jonas et al., the anaesthesiologist intervened in 29 cases (2.9%), mainly for hypertensive surge^33^. The largest cohort was published by Zakrzewski et al. Out of 15,440 surgeries performed under the supervision of "registered respiratory therapists", the intervention of an anaesthesiologist was required for 395 procedures (2.6%)^8^. Unlike our protocol however, patients received intravenous sedation in most cases.

Only a few French publications relate to protocols without an anaesthesiologist present in the operating room (anaesthetic intervention: Batta et al. 2014a^11^: 10/124 (8%); Bouvet et al. 2015^12^: 20/575 (3.4%); Jacquens et al. 2020^34^: 80/1,592 (5%)). Pepin et al. in their series of 248 cataract surgeries performed under topical anaesthesia, did not find any statistically significant difference in terms of intraoperative pain, surgical complications or postoperative visual acuity between patients operated on with and without an anaesthesiologist present^13^.

Koolwijk et al., in the Netherlands, described a protocol similar to ours on a retrospective series of 6,961 cataract surgeries in an independent outpatient centre, under topical anaesthesia and with minimal intraoperative monitoring (heart rate and oxygen saturation) without the following: pre-anaesthetic consultation, anaesthesiologist in the operating room, venous line^35^. They reported 3 emergency team interventions (0.04%) for postoperative vasovagal collapse and no hospitalization was necessary. Murray et al., in a large retrospective series of 6,661 surgeries, performed without anaesthetic supervision over an 8-year period, described only one serious adverse event^22^. They concluded that the majority of cataract surgery procedures under local anaesthesia could be performed safely without the presence of an anaesthesiologist.

### Adverse events and intraoperative complications

Intraoperative pain is the most frequently reported adverse event in the literature with surgery under local anaesthesia^5,11,16,18^. The pain experienced is also a determining factor in patient satisfaction^18^. Rocha et al. reported minimal or moderate intraoperative pain in 13% of cases^5^, which is higher than our results (0.6%). The systematic addition of intracamerular lidocaine to numb the iris has been shown to be effective in achieving additional analgesia and may partly explain this good result^3^. The mean intraoperative pain evaluated in our study was less than 1/10, indicating minimal pain, acceptable to the patient, and 90% of subjects found the intervention painless.

The most common intercurrent event found in our study was agitation/anxiety which was successfully managed by providing tactile and verbal reassurance. In fact, no surgical complications were noted in patients who exhibited anxiety. Some teams^5–8^ use intravenous sedation in case of patient agitation, but systematic venous line and monitoring by an anaesthesiologist would then legally be required in France in that case, which was not possible in our protocol. Placing a venous line can be a painful and stressful experience for patients, especially in the elderly whose veins can be fragile^36^. Additionally, Katz et al. demonstrated, in their study of nearly 20,000 procedures, a significant increase in the frequency of intraoperative adverse events with intravenous sedation^6^. The complication rate was 0.13% with pure topical anaesthesia, 0.78% with locoregional anaesthesia, and it increased to 1.2% when intravenous sedation was combined with topical anaesthesia or locoregional anaesthesia. On the other hand, several publications have shown that low doses of oral anxiolytic can be used without risk to effectively reduce pre- and intraoperative anxiety^5,35,37^.

A surge in hypertension during surgery is one of the most common reason for intraoperative anaesthesiologist intervention, occurring in 0.5 to 5%^5–8,10,11,33^. High blood pressure was found in almost half of our patients (47%). Yet, almost all of our patients had satisfactory blood pressure from the start (96%) which can be explained by the adherence to the usual medication intake and the absence of preoperative fasting.

Posterior capsular rupture with or without vitreous loss can cause major operating difficulties, compromise visual outcome and is considered as the main parameter to assess safety^25,26^. The incidence of posterior capsular rupture ranges between 0.5 and 5.1%^5,11,25,26,35,38^ and has been shown to be correlated with the surgeon's experience^26^. In our study, experienced hospital practitioners, specialist assistants and residents performed the procedures and the rate of posterior capsular rupture was low (0.5%).

### Patient satisfaction

The measurement of patient satisfaction is an interesting tool for evaluating the quality of care and, by taking into account the patient experience, improving practices^39^. Fung et al., 2005^18^, and Batta et al., 2014^11^, reported respectively an ISAS score of 5.6 and 5.46 which is consistent with our findings of 5.66. The factors influencing patient satisfaction are known to be pain, anxiety, surgeon’s skills, but also duration of care^4,18^. The patient track in the CACC is intended to be rapid and optimized to reduce waiting times, a potential source of stress and annoyance, to the lowest level. The average duration of overall management between admission and return home was 2 h which was certainly appreciated by the patients.

### Demographic and economic aspects

The CHB is located in the heart of the Cher area, a medical desert ranked 96^th^ among the 109 French areas in terms of ophthalmologist density. The population of the Cher area is older than the national average and the demand for cataract surgery is high. In a previous study, we demonstrated that the CACC had a positive local epidemiological impact by increasing attractiveness and decreasing leakage of patients to other territories without additionnal costs compared to the conventional operating room (Supplemental file 2)^14^.

### Limitations

The current study has certain limitations. Due to the absence of blood pressure measurement during the operation, any hypertensive surges could not be detected, although this is an adverse event frequently reported in the literature, which may require the intervention of the anaesthesiologist. Further comparative studies are needed to address the role of intraoperative blood pressure measurement as it is not yet known if it can improve safety and outcomes. Yet, the incidence of adverse events remained low and similar to the findings reported by Koolwijk et al.^35^. Visual disturbances were experienced by patients immediately after surgery, and they often had difficulty filling out the questionnaires on their own. The nurse in the monitoring room routinely offered to help patients, which may have influenced their responses. However, this bias was also present in other studies^11,16,18^. This study was performed in an ambulatory surgery centre located within a public general hospital and thus further evaluation is needed for private practice.

This study shows that cataract surgery can be performed safely with a high degree of patient satisfaction in an outpatient setting under topical anaesthesia, without perioperative anaesthetic care or venous line, under minimal intraoperative monitoring and without an anaesthesiologist immediately available. Preoperative screening is essential, but it appears that a large majority of patients can benefit from this type of procedure. Due to the low rate of adverse events and the absence of serious medical complications during cataract surgery under topical anaesthesia, monitoring by an anaesthesia team does not appear essential. An emergency medical team must, however, be reachable and available to respond rapidly if necessary. The protocol used at the CACC offers safe and satisfactory care for the patient, without increasing costs. Surgical activity, however, depends on the organizational specificities of each healthcare establishment and on the motivation of the surgical and anaesthesia teams.

## Supplementary Information

Supplementary File 1.

Supplementary File 2.

Supplementary Legends.

## Data Availability

The datasets generated during and/or analysed during the current study are available from the corresponding author on reasonable request.
