# Author Correction: Ambulatory cataract surgery centre without perioperative anaesthesia care: a prospective cohort study

**DOI:** 10.1038/s41598-021-95473-x

**Published:** 2021-08-09

**Authors:** Quentin Duroi, Jean‑Marie Baudet, Maxime Bigoteau, Malek Slim, Tiphanie Pichard, Pierre‑Jean Pisella, Raoul Kanav Khanna

**Affiliations:** 1 Department of Ophthalmology, Centre Hospitalier Universitaire Régional de Bretonneau, Bretonneau University Hospital of Tours, 2 Boulevard Tonnellé, 37000 Tours, France; 2 Department of Ophthalmology, Centre Hospitalier Jacques Coeur, Bourges, France; 3 INSERM 1253 iBrain «Neurogénomique & Physiopathologie neuronale», Tours, France

Correction to: *Scientific Reports* 10.1038/s41598-021-87926-0, published online 15 April 2021

The original version of this Article contained errors.

In the Materials and methods section, under the subheading ‘Data collection’,

“With the maximum score being 6, satisfaction is considered high for a score above 5.4^18^.”

now reads:

“With the maximum score being 6, satisfaction is considered high for a score above 5.4^18^. This copyrighted questionnaire is the property of Franklin Dexter and the University of Iowa Research Foundation.”

Additionally, in the legend of Figure 2,

"ISAS, Iowa Satisfaction with Anaesthesia Scale^15^.”

now read:

"ISAS, Iowa Satisfaction with Anaesthesia Scale^15^ (copyrighted by Franklin Dexter and the University of Iowa Research Foundation)."

The original Article has been corrected.

