# Supplementary figures and images for: Ambulatory cataract surgery centre without perioperative anaesthesia care: a prospective cohort study
Source: Sci Rep. 2021 Apr 15;11:8311. doi: 10.1038/s41598-021-87926-0 (PMC8050067; doi:10.1038/s41598-021-87926-0)

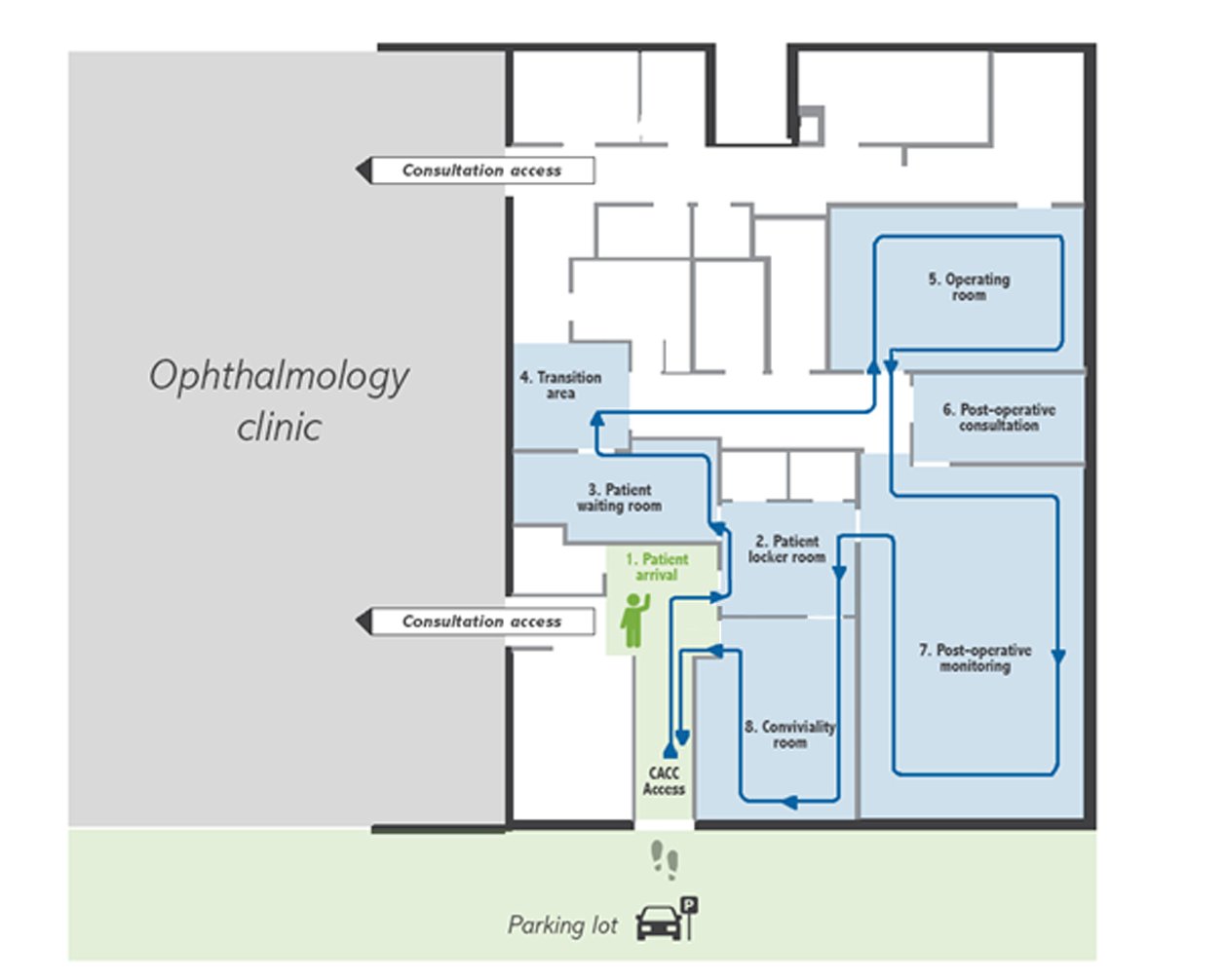

Supplement: Supplementary file 1 [file 41598_2021_87926_MOESM1_ESM.tif]

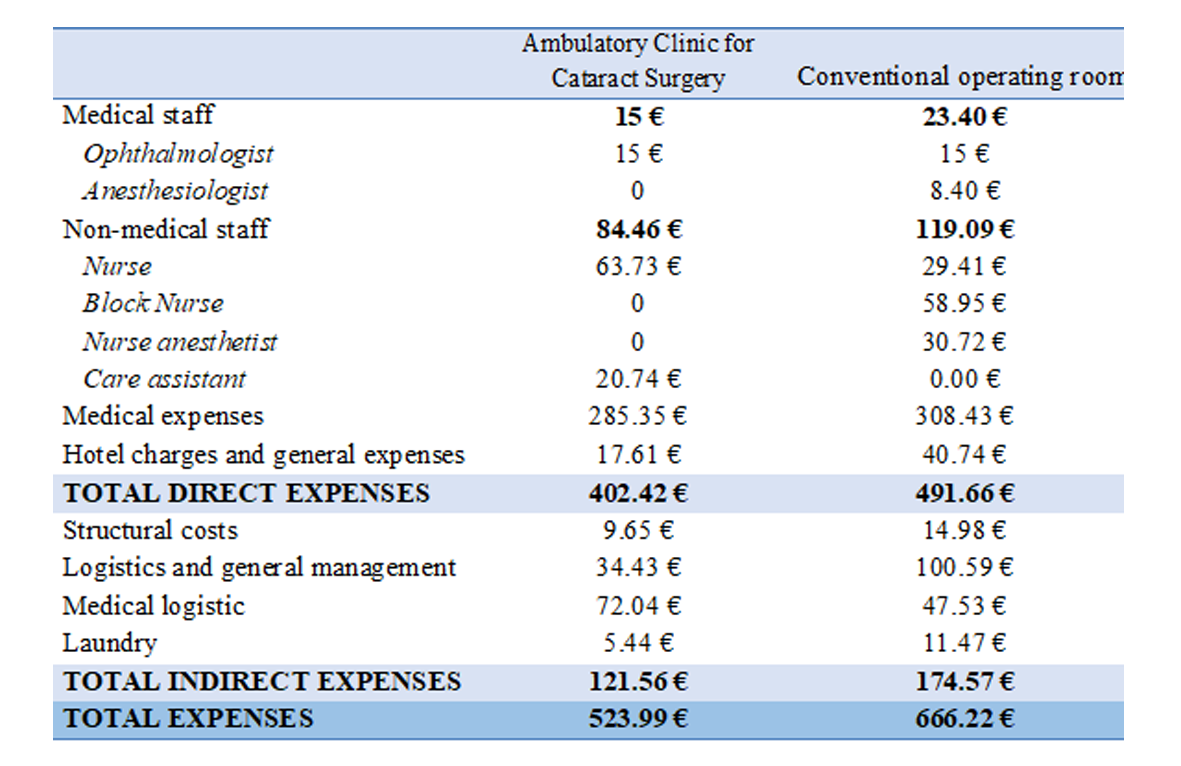

Supplement: Supplementary file 2 [file 41598_2021_87926_MOESM2_ESM.tif]
