# Supplementary material for: Ambulatory cataract surgery centre without perioperative anaesthesia care: a prospective cohort study
Source: Sci Rep. 2021 Apr 15;11:8311. doi: 10.1038/s41598-021-87926-0 (PMC8050067; doi:10.1038/s41598-021-87926-0)
Supplement: Supplementary file 3 — Supplementary Legends. [file 41598_2021_87926_MOESM3_ESM.docx]

**SUPPLEMENTAL FILES**

**Supplemental file 1.** Patient track in the CACC (realized by MB).

**Supplemental file 2.** Unit cost per cataract surgery.
